# Proliferative retinopathy as a feature of Vogt Koyanagi Harada Disease: a report of two cases

**DOI:** 10.1186/s12886-020-01736-y

**Published:** 2020-12-01

**Authors:** Moustafa S. Magliyah, Abdulmajeed S. Al-Fakhri, Hassan A. Al-Dhibi

**Affiliations:** Vitreoretinal Division, King Khaled Eye Specialist Hospital, Al- Oruba Street, PO Box 7191, Riyadh, 11462 Kingdom of Saudi Arabia

**Keywords:** Pre-retinal hemorrhage, Proliferative retinopathy, Retinal neovascularization, VKH, Case report

## Abstract

**Background:**

Proliferative retinopathy is an uncommon feature of Vogt Koyanagi Harada (VKH) disease which might indicate poor uveitis control in these patients. We aim to describe the clinical features and outcome of management of proliferative retinopathy in 2 patients with VKH.

**Case Presentation:**

19 and 33 years old females with VKH presented with unilateral proliferative retinopathy. Both patients had neovascularization of the optic disc (NVDs) and one patient had neovascularizations elsewhere (NVEs) and preretinal hemorrhage. Both patients had exudative retinal detachments (ERD). Systemic steroids and immunomodulatory agents were successfully used to control inflammation and achieve regression. One patient developed fibrous tissue formation at the disc area as well as an epiretinal membrane formation, for which she had pars plana vitrectomy with membrane peeling. Both patients had controlled inflammation with stable vision.

**Conclusions:**

Proliferative retinopathy can present variably in VKH patients and indicates persistent inflammation which is incompletely controlled. Proper uveitis control is sufficient to achieve regression of retinal neovascularization.

## Background

Vogt–Koyanagi–Harada (VKH) disease is a bilateral granulomatous panuveitis which leads to exudative retinal detachments (ERD) [1]. Extraocular manifestations of VKH include meningismus, tinnitus, dysacusia, vitiligo, alopecia and poliosis [2–4]. Based on timely initiation of immunosuppressive treatments, it is classified to acute and chronic recurrent subtypes [5]. Chronic recurrent VKH is characterized by vitiligo, recurrent anterior uveitis, and sunset glow fundus [6]. Neovascularization of the optic disc (NVD) was reported in two cases of VKH in the pre-immunomodulation therapy era [7, 8]. Here we present two cases of chronic recurrent VKH presented with proliferative retinopathy. Both cases were diagnosed based on the revised diagnostic criteria for Vogt-Koyanagi-Harada disease [9].

## Case Presentation

### Case 1

**Fig. 1:**
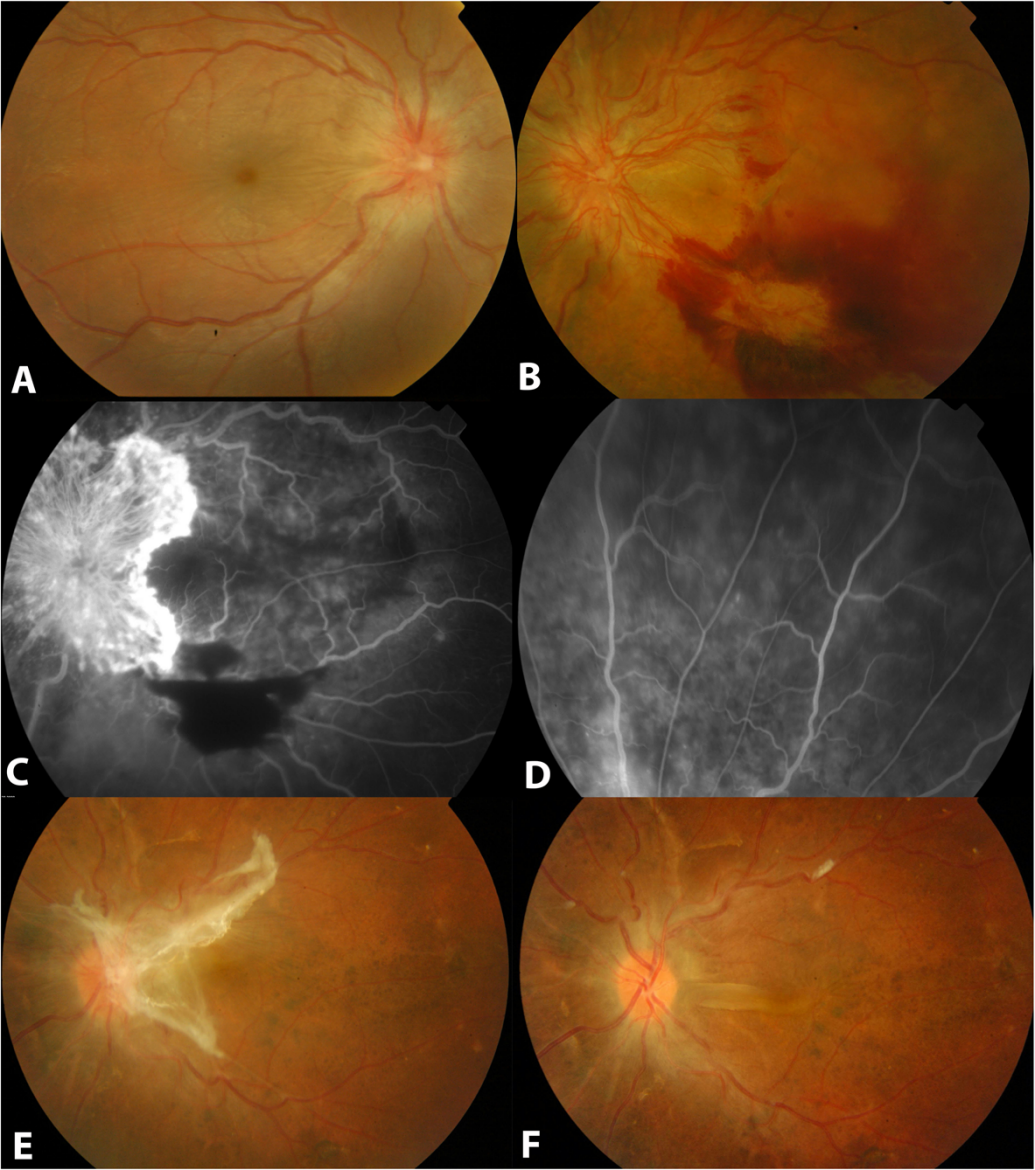
Clinical and Ancillary Findings of proliferative retinopathy in a 19 years old female (Case 1) with Vogt Koyanagi Harada (VKH). **a** is a fundus photo of the right eye showing a hyperemic optic disc hyperemia with an exudative retinal detachment (ERD). **b** fundus photo of the left eye showing neovascularization of the optic disc (NVD) with an intraretinal hemorrhage. **c** is a Fundus fluorescein angiography (FFA) of the posterior pole in the left eye showing fluorescein leakage from the NVDs and blockage at the area of retinal hemorrhage. **d** is an FFA of the peripheral retina in the left eye showing absence of retinal capillary non-perfusion. **e** is a fundus photo of the left eye showing regressed NVDs after proper uveitis control was achieved, leaving a fibrous tissue at the disc area and an epiretinal membrane. **f** is a fundus photo of the left eye showing resolution of the fibrous tissue and epiretinal membrane following pars plana vitrectomy with membrane peeling. Note the pigmentary retinal changes indicating chronic VKH changes

A 19-year-old female was referred to our hospital 8 years ago as a case of bilateral granulomatous panuveitis with bullous ERD in both eyes associated with had headaches and tinnitus. She was diagnosed as incomplete VKH and was started on oral steroids 75 mg/day. One month later, her vision improved to 20/40 in both eyes with flat retina in both eyes and trace anterior chamber cells. She lost her follow ups for one year, after which she presented with decreased vision in both eyes. Best corrected visual acuity (BCVA) of 20/200 in the right eye and 20/400 in the left eye with normal intraocular pressure (IOP). Anterior chamber (AC) was deep in both eyes with 3 + cell. Fundus examination showed exudative retinal detachment with hyperemic optic discs in both eyes, with the left eye having NVDs with preretinal hemorrhages (Fig. 1a and b). Fundus fluorescein angiography (FFA) revealed leakage from optic disc margins in the right eye, while showed fluorescein leakage from the NVDs in the left eye and blockage at the area of retinal hemorrhage (Fig. 1c). Peripherally, absence of capillary nonperfusion areas was noted on FFA (Fig. 1d). Uveitis systemic workup including complete blood count (CBC), renal function test (RFT), liver function test (LFT), chest computed tomography (CT), fluorescent treponemal antibody absorbed (FTA-Abs) and purified protein derivative (PPD) and TB-Quantiferone Gold test was negative. The impression was VKH and the patient was given 3 doses of 1 g intravenous (IV) methylprednisolone daily followed by oral prednisolone 75 mg tapering dosage and oral azathioprine 50 mg twice daily. One year later, regression of NVDs in the left eye resulted in fibrous tissue formation around the disc with epiretinal membrane formation (Fig. 1e). Pars plana vitrectomy with membrane peeling resulted in resolution of the fibrous tissue and epiretinal membrane (Fig. 1f). Over 5 years of follow ups, she maintained a BCVA of 20/25 in the right eye and 20/40 in her left eye with quite anterior and posterior segments.

### Case 2

**Fig. 2:**
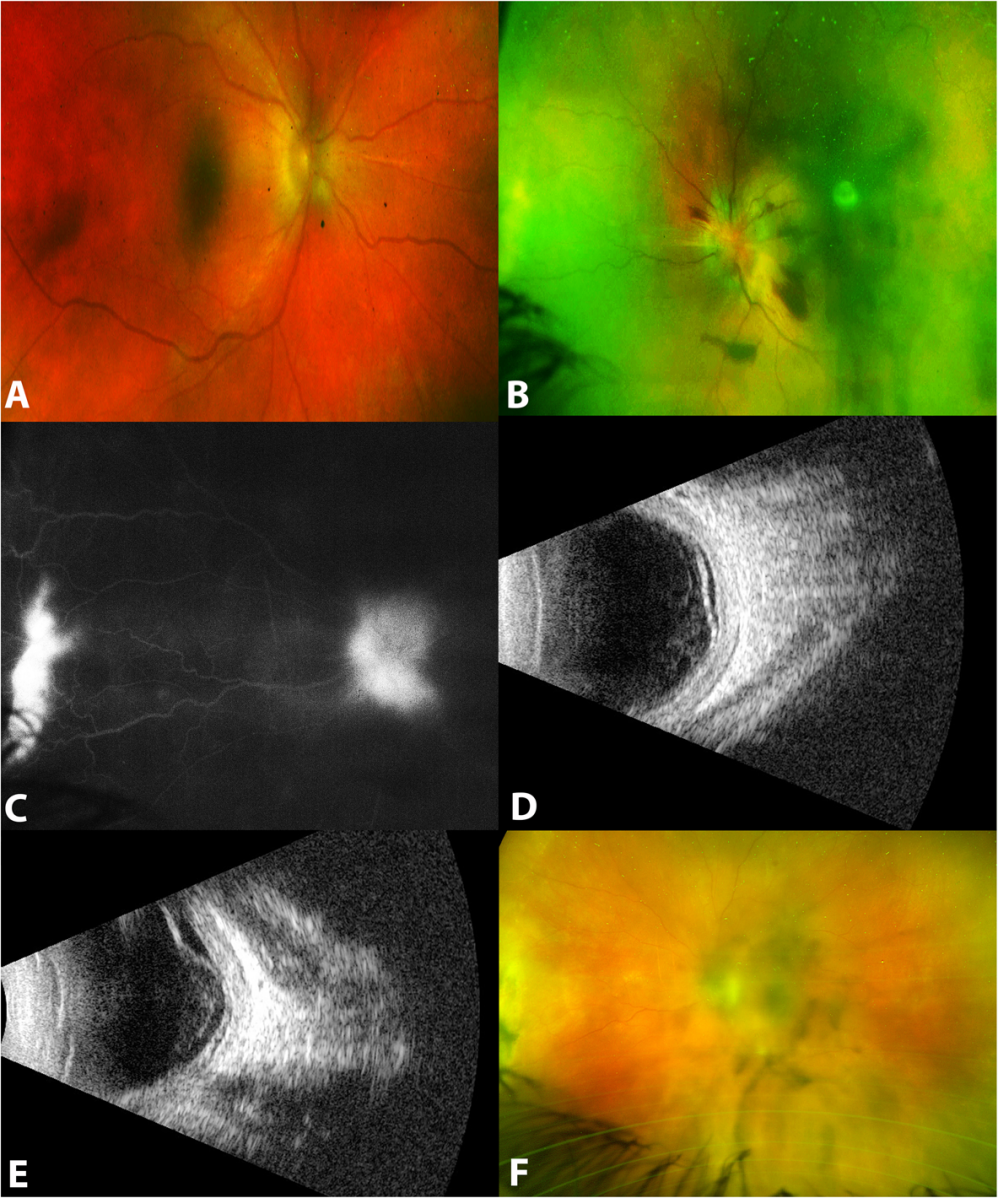
Clinical and Ancillary Findings of proliferative retinopathy in a 33 years old female (case 2) with Vogt Koyanagi Harada (VKH). **a** is a fundus photo of the right eye showing a shallow exudative retinal detachment (ERD) with a hyperemic disc. **b** is a fundus photo of the left eye showing optic disc neovascularization (NVD), nasal neovascularization elsewhere (NVE), pre-retinal and vitreous hemorrhages causing hazy media. **c** is a fundus fluorescein angiography of the left eye showing leakage from the NVDs and NVE. **d** and **e** are B scan ultrasonographic examinations showing shallow ERD in the right and left eyes, respectively. **f** is a fundus photo of the left eye showing regression of retinal neovascularization and resolution of the pre-retinal and vitreous hemorrhages, as well as the development of posterior subcapsular cataract

A 33 years old female who is a known case of type 1 diabetes mellitus for 9 years was referred to our hospital 2 years ago as a case of recurrent VKH 6 months following initial presentation. She reported to have a history of headache and tinnitus in association with ophthalmic manifestations. Upon her presentation, she was on 15 mg oral steroids. Ophthalmic examination revealed BCVA of 2/300 in the right eye and hand motion in the left eye, and IOP of 9 and 7 mmHg. Both eyes had 3 + cells in the AC and 3 + vitritis. Posterior segment examination showed macular involving ERD and hyperemic disc were found in the right eye (Fig. 2a), while in the left eye, there were NVDs and nasal neovascularizations elsewhere (NVEs) associated with pre-retinal and vitreous hemorrhages which precluded further assessment of the retina (Fig. 2b). FFA showed bilateral disc leakage and leakage from NVDs and NVEs in the left eye (Fig. 2c). B scan confirmed ERDs in both eyes (Fig. 2d and e). Uveitis systemic workup including CBC, RFT, LFT, chest CT, FTA-Abs, PPD and TB-Quantiferone Gold test was unremarkable. Her Hemoglobin A1C was 7.6% (reference range is 5.7 – 6.4%). Human Leukocyte Antigen (HLA) typing revealed that the patient carried the HLA-DRB1*03 (DR3), HLA-DRB*04 (DR4) and HLA-DQB1*02 (DQ2). She was diagnosed as incomplete VKH and was given 3 doses of 1 g IV methylprednisolone daily followed by oral prednisolone 1 mg/kg/day tapering dosage and oral mycophenolate mofitel 1 g twice daily. One month later, she presented with controlled inflammation and persistent pre-retinal hemorrhage for which she had a single dose of intravitreal ranibizumab injection. After one month, resolution of pre-retinal hemorrhage along with regression of retinal neovascularizations were observed (Fig. 2f), along with the development of posterior subcapsular cataracts in both eyes. Her vision was improved to 20/40 in both eyes. Over 1 year follow up, she had maintained her vision and had quite eyes.

## Discussion and Conclusions

Although retinal neovascularization is a known feature in uveitic entities like Behçet’s disease, juvenile idiopathic arthritis, sarcoidosis, pars planitis, Crohn’s disease and systemic lupus erythematosus [9], it is an uncommon feature of VKH. Only two VKH cases associated with NVDs were found in the literature. Table 1 summarizes all VKH patients who manifested proliferative retinopathy [7, 8]. Notably, all four patients were females, young or middle aged, and had granulomatous panuveitis with ERD. Duration between presentation and development of retinal neovascularization ranged between 6 months and 1 year. All patients were either on low dose or discontinued systemic steroids treatment. Systemic uveitis treatment resulted in regression of retinal neovascularization in three patients. The cause of proliferative retinopathy in VKH is not clear, but it seems to be related to long durations of either treatment discontinuation or suboptimal dosage. Although the second patient has type 1 diabetes, proliferative retinopathy was found in only one eye. No signs of diabetic retinopathy were found in the contralateral eye on fundus examination and FFA. Regression of retinal neovascularization upon initiation of systemic immunosuppression in association with a single dose of anti-vascular endothelial growth factor (Anti-VEGF) without the need for peripheral laser photocoagulation application further confirm the prominent role of persistent uveitis in the development of retinal neovascularization in this patient. Although Anti-VEGF injection was found to be effective in treating proliferative diabetic retinopathy in DRCR.net Protocol S, intravitreal injections were performed on monthly basis to control proliferative diabetic retinopathy [10]. Furthermore, only 19% of eyes achieved complete regression of neovascularizations after a single Anti-VEGF injection. Although the severity of proliferative diabetic retinopathy was not detailed for eyes which responded rapidly to Anti-VEGF, it is unlikely to achieve such rapid response in advanced clinical picture with pre-retinal and vitreous hemorrhages like our patient. Only few cases have been reported the association between VKH and type 1 diabetes mellitus in the literature, including two cases from Saudi Arabia [11–15]. HLA types found in patients with this disease association included DR4, DQ4, DR9 and DQ3. In our second patient, HLA types found included DR3, DR4 and DQ2. These findings help to expand the knowledge about HLA types found in patients with VKH in association with type 1 diabetes mellitus. The finding of HLA-DR4 which is strongly associated with VKH is further suggestive of the diagnosis [16].

**Table 1:** Summary of all documented Vogt Koyanagi Harada patients who developed proliferative retinopathy

Patient	Demographics	Initial VKH signs	Initial treatment	Time to develop proliferative retinopathy	Signs of proliferative retinopathy	Treatment	Outcome
1	22 years old African female	AC cells, hyperemic discs and ERD in both eyes	Oral prednisolone 100 mg every other day	One year	NVDs in both eyes	N/A	N/A
2	46 years old Indian female	AC cells with ERD in both eyes	80 mg prednisolone daily	8 months	NVDs in the left eye	Oral steroids	Regressed NVDs
3	19 years old Saudi female	bilateral granulomatous panuveitis with bullous ERD in both eyes	75 mg/daily	One year	NVD in the left eye with preretinal hemorrhages	Oral steroids, Azathioprine, PPV + MP	Resolved fibrous tissue and ERM, controlled inflammation
4	33 years old Saudi female	N/A	N/A	6 months	NVDs, NVE with pre-retinal hemorrhages in the left eye	Oral steroids, mycophenolate mofitel, intravitreal Ranibizumab	Regressed NVDs and NVEs, resolved pre-retinal hemorrhages and controlled inflammation

*AC* Anterior Chamber, *ERD* Exudative retinal detachment, *ERM* Epiretinal membrane, *MP* Membrane peeling, *PPV* Pars plana vitrectomy, *N/A* Not available, *NVD* Neovascularization of the optic disc, *NVE* Neovascularization elsewhere, *VKH* Vogt Koyanagi Harada

Extended period of uncontrolled inflammation is also thought to be associated with retinal neovascularization in other uveitis entities [17]. Although peripheral retinal capillary non perfusion could not be clearly appreciated in the second patient who had vitreous hemorrhage, the absence of capillary nonperfusion in the first patient decreases the likelihood of retinal ischemia as a sole factor for development of retinal neovascularization. Similar findings were observed in other uveitic entities as well, indicating an important role of prolonged inflammation in pathologic vasculogenesis [17]. Proinflammatory cytokines including IL-1b and TNF-a, can promote endothelial proliferation and pathologic angiogenesis [18]. Both IL-1b and TNF-a were found to be elevated in VKH disease [19]. Prolonged duration of inflammation prior to the development of retinal neovascularization, as well as regression of the new vessels upon proper uveitis control support the role of inflammation in proliferative retinopathy in uveitis patients. Anti-VEGF was used in the second patient as an adjunct treatment to inhibit further neovascularization prior to the complete response to systemic immunosuppression and prevent further progression of proliferative retinopathy [20]. Pars plana vitrectomy with membrane peeling can be used to address the fibrous tissue from regressed NVDs and epi-retinal membrane once proper control of inflammation is achieved.

In conclusion, Proliferative retinopathy is an uncommon feature of VKH, especially in patients with suboptimal control of their inflammation. Proper uveitis management is crucial to achieve regression of retinal neovascularization.

## Data Availability

Data sharing is not applicable to this article as no datasets were generated or analyzed during the current study.

## Abbreviations

AC: Anterior chamber
Anti-VEGF: Anti-vascular endothelial growth factor
BCVA: Best corrected visual acuity
CBC: Complete blood count
CT: Computed tomography
ERD: Exudative retinal detachments
FFA: Fundus fluorescein angiography
FTA-Abs: Fluorescent treponemal antibody absorbed
IOP: Intraocular pressure
IV: Intravenous
LFT: Liver function test
mg/kg/day: Milligrams per kilograms per day
NVDs: Neovascularizations of the optic disc
NVEs: Neovascularizations elsewhere
PPD: Purified protein derivative
RD: Retinal detachment
RFT: Renal function test
VKH: Vogt Koyanagi Harada
